# Modified electroconvulsive therapy for treatment-resistant self-injurious behavior in autism spectrum disorder with severe intellectual disability: a case report

**DOI:** 10.3389/fpsyt.2026.1800695

**Published:** 2026-06-17

**Authors:** Ping Xu, Didi Ding, Xuehua Han, Junjun Liu, Hao Tang

**Affiliations:** 1 Department of Psychiatry, Nanjing Lishui District Psychiatric Hospital, Lishui, China; 2 Department of Psychiatry, The Third People’s Hospital of Lishui District, Nanjing, China; 3 Department of Psychiatry, Nanjing Meishan Hospital, Nanjing, China; 4 Department of Psychiatry, Medical College of Soochow University, Suzhou, China; 5 Suzhou Guangji Hospital, The Affiliated Guangji Hospital of Soochow University, Suzhou, China; 6 Department of Psychiatry, The Affiliated Brain Hospital of Nanjing Medical University, Nanjing, China

**Keywords:** autism spectrum disorder, case report, comorbid, intellectual disability, modified electroconvulsive therapy, self-injurious behavior

## Abstract

**Background:**

Treatment options for severe self-injurious behavior (SIB) in autism spectrum disorder (ASD) with comorbid intellectual disability (ID) are limited.

**Case presentation:**

A 24-year-old man with ASD and severe ID presented with treatment-refractory SIB (an 11-year history; baseline frequency of 5–6 hand-biting episodes per day during pre-modified electroconvulsive therapy (MECT) hospitalization observation), despite multiple pharmacotherapy trials. On Day 27, baseline assessments and pre-MECT demonstrated a Violence Risk Assessment Scale (VRAS) score of 18, a Brief Psychiatric Rating Scale (BPRS) score of 74, an Activities of Daily Living (ADL) score of 46, and 4–5 h of nocturnal sleep each night. After 12 sessions of MECT administered over 7 weeks, the primary outcome, that is, SIB frequency, completely resolved (no hand-biting episodes each day). Additionally, secondary outcome measures showed marked improvement, including a VRAS score of 7 (a 61.1% reduction), a BPRS score of 47 (a 36.5% reduction), an ADL score of 34 (a 26.1% improvement in functional independence), and 5–6 h of nocturnal sleep each night. At 30 days post-MECT (Day 106, during continued hospitalization), therapeutic gains were sustained and further enhanced: the VRAS score persisted at 7, the BPRS score decreased to 35 (a 52.7% reduction from baseline), and the ADL score improved to 30 (a 34.8% improvement in functional independence from baseline), with complete resolution of SIB (self-directed harmful acts) and aggressive behavior (other-directed harmful acts). The patient scored zero on the VRAS. No serious adverse events were observed during hospitalization.

**Conclusion:**

Based on controlled nursing frequency counts, mental assessment scales, and photographic wound records, this case provides preliminary observational evidence that MECT may reduce treatment-refractory SIB in patients with ASD and severe ID. Further controlled studies using standardized SIB outcome measures are warranted, given the lack of validated SIB-specific instruments and the limitations inherent to a single-case design.

## Background

While intellectual disability (ID) involves substantial deficits in both intellectual and adaptive functioning, autism spectrum disorder (ASD) is a neurodevelopmental disorder that begins during the developmental period and is characterized by impaired social communication, repetitive behaviors, and restricted interests (1). ASD and ID frequently co-occur. Comorbid ID is present in 37.9% of children with ASD (2); conversely, ASD is prevalent in 18.04% of individuals with ID (3). This comorbidity is associated with more severe self-injurious behavior (SIB), poorer verbal communication, and substantial caregiving burden (4, 5).

Self-injurious behavior (SIB) is defined as repetitive, self-directed behavior that results in bodily harm, including self-hitting, biting, scratching, hair pulling, and other self-damaging acts (6). Aggressive behavior refers to intentional acts, such as hitting, kicking, or biting others or damaging property, that are directed at other people or objects and result in harm or damage (7). Despite being separate behavioral manifestations, SIB and aggressive behavior frequently co-occur in individuals with developmental disabilities. With prevalence rates of 4.9% in ID (8) and 42% in ASD (9), SIB poses a major clinical challenge in these populations. Patients with ASD-ID comorbidity often exhibit more severe symptoms, including high-frequency, high-intensity SIB that can result in moderate-to-severe injuries (10). Fong et al. (11) reported that among 30 children with severe SIB, 93.3% met the ASD criteria, whereas more than 90% had psychiatric or medical comorbidities. Beyond direct physical trauma, SIB substantially increases the risk of psychiatric complications, caregiver burnout, and psychiatric hospitalization (12), profoundly impairing both patient and family functioning.

Managing severe SIB in patients with ASD remains challenging. In pharmacotherapy-refractory cases, FDA-approved antipsychotics (risperidone and aripiprazole) demonstrate limited efficacy, and metabolic adverse effects restrict their long-term use (13). Behavioral interventions also yield variable outcomes in patients with severe ID due to cognitive and communication barriers. In a comprehensive review, Wachtel et al. proposed that SIB in ASD may represent the clinical manifestation of agitated catatonia. Moreover, it was suggested that modified electroconvulsive therapy (MECT) may alleviate SIB by targeting catatonic symptoms, with favorable tolerability, supporting the potential safety and efficacy of MECT for refractory SIB in ASD (14). Vaquerizo-Serrano et al. conducted a systematic review and meta-analysis of 12 studies (n = 1,534). SIB was present in 27.7% to 90.9% of patients with ASD and comorbid catatonia, and the response to benzodiazepines was inconsistent. In contrast, MECT was associated with marked improvement in catatonic symptoms (15). Smith et al. conducted a retrospective analysis of 32 patients with ASD and/or ID treated with MECT. Notably, 94% achieved a positive clinical response (CGI-I ≤3), with significant pre- to post-treatment improvement (p < 0.001) and no major adverse events. These findings support the safety and tolerability of MECT for SIB in this population (16). However, systematic longitudinal data on this patient population remain scarce.

Despite the emerging interest in MECT for neurodevelopmental disorders, existing reports lack comprehensive documentation that combines standardized psychiatric assessments, quantitative behavioral data, and objective clinical evidence. To address this gap, this case report presents multidimensional longitudinal data on the efficacy and safety of MECT. The primary outcome measure was the frequency of SIB episodes, whereas the secondary outcome measures included validated psychiatric rating scales (VRAS, BPRS, and ADL), which assess behavioral risk, general psychopathology, and daily functioning, respectively.

## Case presentation

### Presenting history

A 24-year-old unmarried, unemployed man with no formal education was admitted on 24 September 2025, with the chief complaint of “delayed language development and social difficulties since childhood, with frequent self-injurious behavior for 11 years.”

### Psychiatric and medical history

After being separated from his family in September 2013, the patient was reunited with them in February 2025, 11.5 years later. During this period, he was consistently diagnosed with ASD while receiving psychiatric care at several institutions. Multiple pharmacological trials at therapeutic doses, including mood stabilizers (valproic acid, lithium carbonate, and oxcarbazepine), antipsychotics (olanzapine and aripiprazole), and clonazepam, demonstrated unsatisfactory responses, with persistent SIB and behavioral dysregulation.

Following family reunification, the patient exhibited severe impairment and required substantial caregiver support. The two core maladaptive behaviors were (1): SIB, which involved hand and knee biting eight to ten times daily at home, resulting in bilateral full-thickness skin breakdown; and (2) impulsive hostility against caregivers occurring five to seven times weekly. These behaviors progressively worsened during the 6 months before hospitalization.

The patient was diagnosed with secondary pulmonary tuberculosis (sputum smear-positive for acid-fast bacilli) in December 2024. He completed a standard 6-month four-drug regimen (isoniazid, rifampicin, pyrazinamide, and ethambutol), with documented sputum conversion to a negative status in June 2025. His prenatal and perinatal histories were unremarkable. There was no family history of psychiatric disorders, intellectual disabilities, or epilepsy.

### Physical and mental status examination

**Figure 1:**
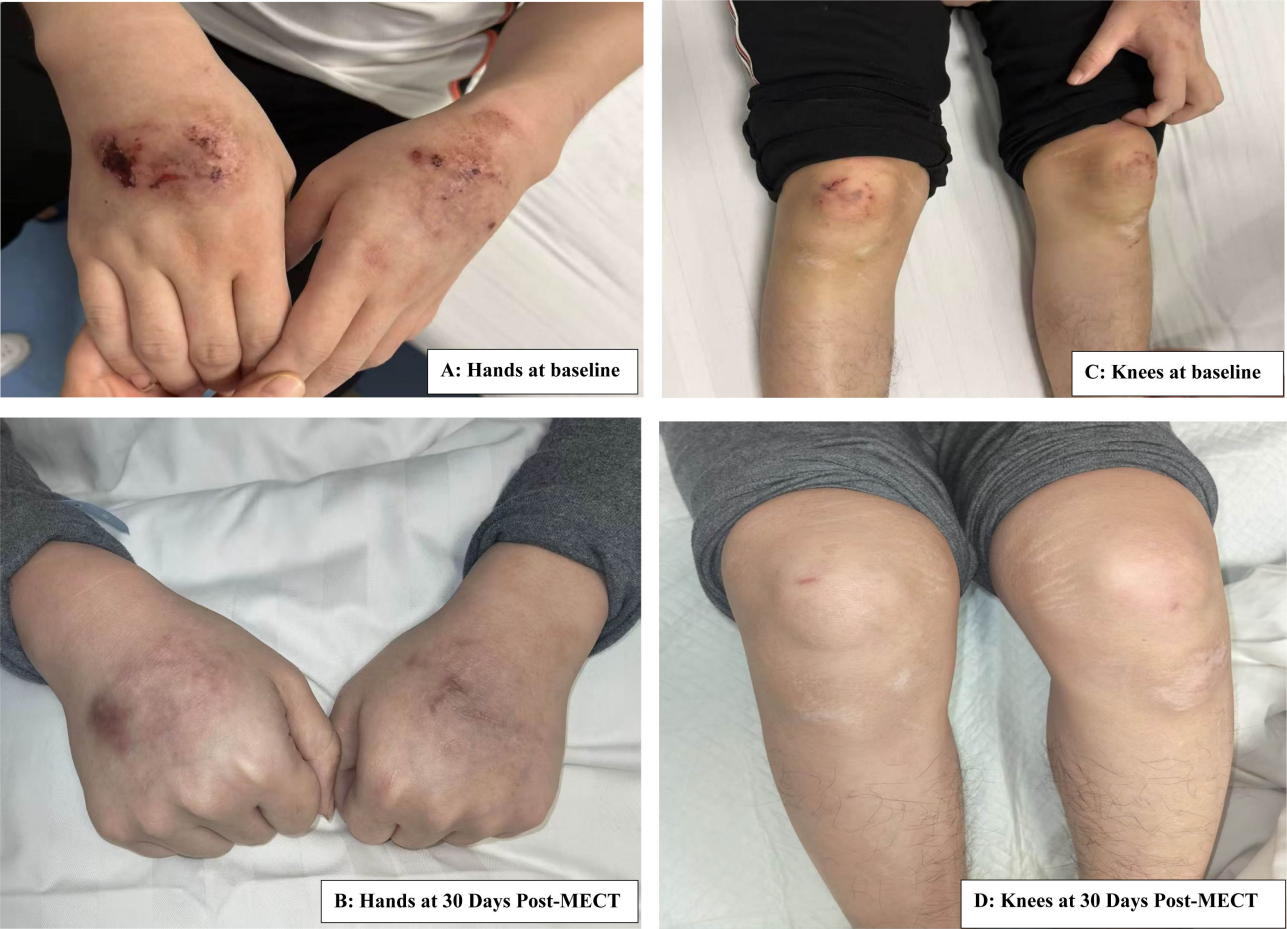
Photographic evidence of improvement in self-injurious behavior. **(A)** Bilateral hands at baseline, showing multiple wounds and scabs; **(B)** Bilateral hands at 30 days after MECT, demonstrating near-complete healing; **(C)** Bilateral knees at baseline, showing severe skin damage; **(D)** Bilateral knees at 30 days after MECT, demonstrating wound resolution. Self-injurious behavior decreased from five to six episodes/day to no episodes/day. Secondary clinical outcomes at the 30-day follow-up are as follows: 61.1% reduction in the VRAS score (18→7), 52.7% reduction in the BPRS score (74→35), and 34.8% improvement in the ADL score (46→30; lower scores indicate greater independence). Written informed consent for image publication has been obtained from the patient’s legal guardian, and de-identification protocols have been maintained.

Physical examination revealed stable vital signs (temperature, 36.2 °C; pulse, 75 bpm; blood pressure, 111/73 mmHg; and body mass index, 19.6 kg/m²). Multiple acute bite wounds (0.5–2.0 cm) on both hands and knees showed partial-thickness tissue loss with active bleeding (**Figures 1A–D**). The results of cardiovascular, respiratory, abdominal, and neurological examinations were unremarkable.

The mental status examination revealed poor eye contact and motor stereotypies (hand-flapping and body rocking). Vocabulary was limited to approximately 30–50 single words (per caregiver report), with echolalic and dysarthric speech. The patient’s affect was labile and irritable, with unpredictable outbursts. The pain response appeared to be diminished (hypoalgesia to noxious stimuli). Judgment and insight were absent.

### Diagnostic investigations

Results from laboratory evaluations, including hepatic and renal function tests, infectious disease screening, thyroid function tests, electrolyte levels, glucose testing, and ultrasound examinations, were all within normal limits (**Supplementary Table 1**). Sputum examination was negative for acid-fast bacilli. Electrocardiogram and electroencephalogram (EEG) results were normal. Chest computed tomography revealed minimal fibrotic strands consistent with prior tuberculosis. Brain magnetic resonance imaging revealed no acute pathology.

Formal assessment using the Wechsler Adult Intelligence Scale–Fourth Edition was not feasible because of severe language impairment. Abbreviated assessment using non-verbal subtests yielded estimated scores within the severe intellectual disability range (estimated intelligence quotient (IQ) < 35). The Childhood Autism Rating Scale score was 42 (severe autism). The Activities of Daily Living (ADL) score was 46 (substantial dependence). The Brief Psychiatric Rating Scale (BPRS) score was 74. The Violence Risk Assessment Scale (VRAS) score was 18 (high risk). On Day 27, baseline observation documented five to six SIB episodes daily, five to seven aggression episodes weekly, three to four physical restraint episodes weekly, and 4–5 h of sleep per night (**Table 1**). SIB episodes were defined as discrete self-biting acts directed toward the patient’s own hands or knees, whereas aggressive episodes were defined as directed physical acts toward staff. Each act was counted as a single episode, and repeated acts were recorded as separate episodes once the preceding behavior had completely stopped. Both behaviors were continuously observed around-the-clock and recorded by nursing staff in a standardized behavioral log. Physical restraints (soft padded limb cuffs) were used only when acute SIB or aggression posed an imminent risk of injury and de-escalation strategies had failed. Each application was time-limited (target duration, 15–30 min) and documented according to institutional protocol, which required physician authorization, continuous monitoring, and post-episode review. During restraint use, nursing staff regularly monitored the patient’s circulation, skin integrity, and comfort. Privacy was maintained at all times, and restraints were removed immediately once the imminent risk had subsided, in accordance with the least-restrictive, shortest-duration principle.

**Table 1:** Frequency of behavioral symptoms at different time points during and after MECT treatment.

Behavioral type	Baseline (Day 27, Pre-MECT)	After 3rd session (Day 30)	After 6th session (Day 37)	After 9th session (Day 55)	After 12th session (Day 76)	30 days post-MECT (Day 106)
Self-injurious behavior (times/day)	5–6	3–5	6–8	0	0	0
Aggressive behavior (times/week)	5–7	3–4	4–5	1–2	0	0
Physical restraint (times/week)	3–4	1–2	3–4	0	0	0
Nocturnal sleep (hours/night)	4–5	3–4	3–4	4–5	5–6	4–5

MECT, modified electroconvulsive therapy; SIB, self-injurious behavior.

Data collection methodology: all behavioral frequencies were documented through standardized 24-hour structured nursing observation using predefined operational criteria. Each data point represents the daily or weekly average frequency recorded during routine clinical monitoring at each assessment time point.

### Diagnosis

The patient met the DSM-5-TR criteria for (1) Autism Spectrum Disorder and (2) Severe Intellectual Disability.

### Initial treatment and MECT decision

**Figure 2:**
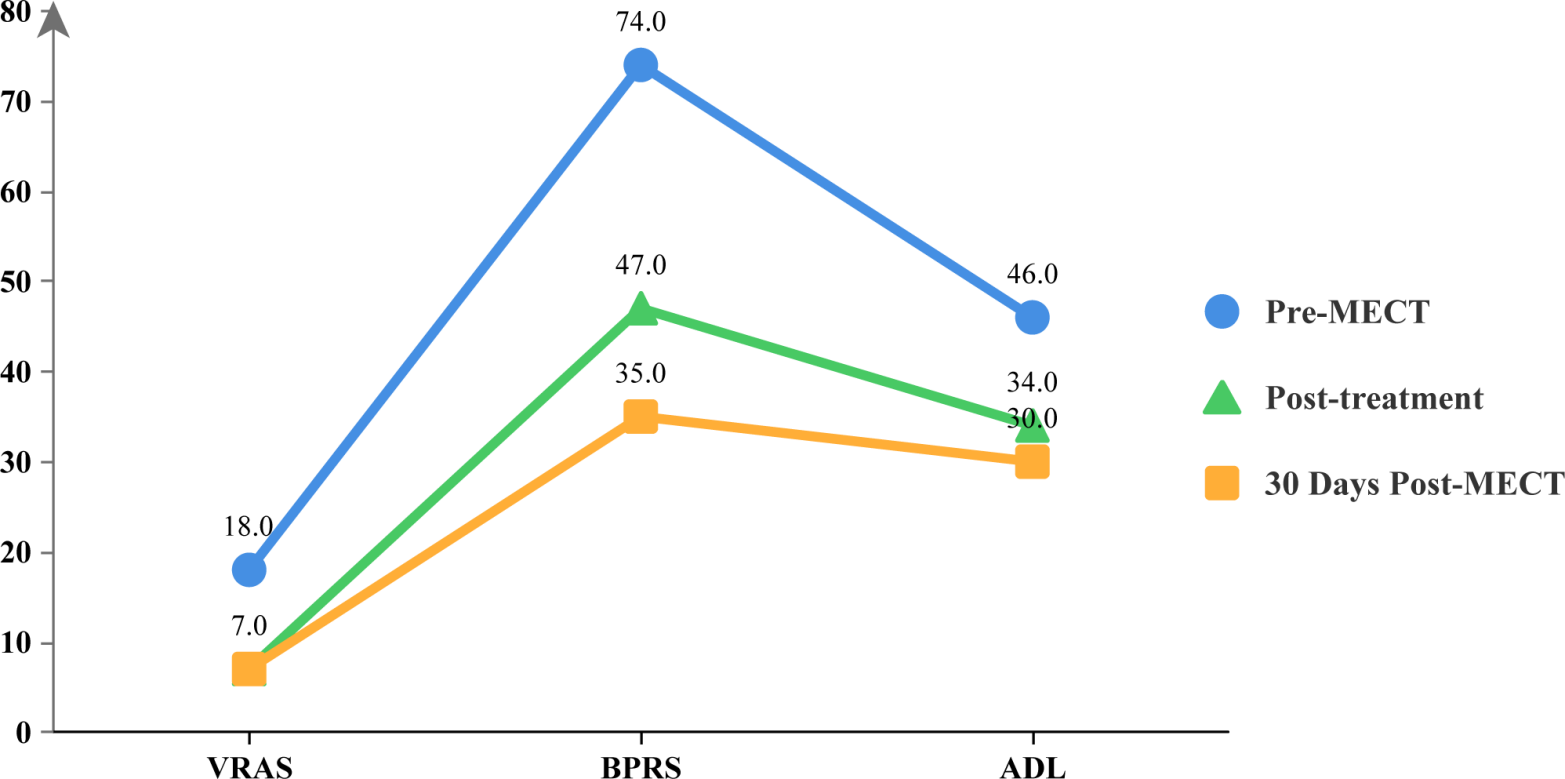
Clinical outcomes across assessment instruments during MECT treatment and follow-up. Longitudinal changes in Violence Risk Assessment Scale (VRAS), Brief Psychiatric Rating Scale (BPRS), and Activities of Daily Living (ADL) scores at baseline (Day 27, pre-MECT), post-treatment (Day 76, after Session 12), and 30 days post-MECT (Day 106). Percentage reductions from baseline to final follow-up: 61.1% in the VRAS score (18→7), 52.7% in the BPRS score (74→35), and 34.8% improvement in the ADL score (46→30). For the ADL scale specifically, lower scores indicate greater functional independence and improved adaptive functioning (score range: 14–56; Pre-MECT: 46 [Day 27] → post-treatment: 34 [Day 76] → 30 days post-MECT: 30 [Day 106]).

Upon admission, 24-h supervision and behavioral management were initiated. Olanzapine (20 mg/day) combined with valproic acid (1,000 mg/day) showed no improvement on Day 10 (VRAS score, 18; SIB frequency, 5–6 episodes/day). The regimen was changed to risperidone (3 mg/day) and valproic acid (1,000 mg/day). On Day 26, persistent refractoriness was recorded (SIB, 5–6 daily episodes; aggression, 5–7 weekly episodes; VRAS score, 18; and BPRS score, 74) (**Figure 2**).

Considering the poor response to two antipsychotics combined with a mood stabilizer and the 11-year history of pharmacotherapy resistance, a multidisciplinary consultation concluded that MECT was potentially beneficial and that no contraindications were present. The pre-MECT evaluation confirmed stable cardiovascular function, resolved tuberculosis, and no contraindications to anesthesia. The Ethics Committee of Nanjing Lishui District Psychiatric Hospital approved MECT after the patient’s legal guardian gave their full informed consent (Approval No. 2025-YLLW-01). Given the patient’s severe intellectual disability (estimated IQ < 35), absent judgment, and markedly limited communicative capabilities, formal assent from the patient could not be obtained; all medical decisions were made by the legal guardian in the patient’s best interest, in consultation with the multidisciplinary clinical team.

### Treatment

#### Treatment protocol

MECT commenced on 21 October 2025 (Day 27), three times weekly using the Thymatron^®^ System IV with bilateral frontotemporal placement. Session 1 used a 25% charge (151.2 mC, 0.9 J) to determine the seizure threshold. Sessions 2–12 used a 50% charge (302.4 mC, 1.8 J) with a 0.5-ms pulse width, frequency of 70 Hz, and duration of 6.0 s. The risperidone dose was adjusted to 2 mg/day, and valproic acid was discontinued during MECT.

After 3 min of preoxygenation, anesthesia was induced with propofol (1.5 to 2.0 mg/kg; 90–120 mg) and succinylcholine (0.5 to 1.0 mg/kg; approximately 40 mg). Atropine (0.5 mg IV) was administered 5 min before induction. Motor seizure duration was monitored using the cuff method, together with EEG monitoring.

#### Treatment course and clinical response

In total, 12 sessions were completed over 7 weeks (21 October–9 December 2025) without any serious adverse events. Motor seizure duration ranged from 28 to 45 s (mean, 35.2 ± 5.8 s), and EEG duration ranged from 35 to 58 s (mean, 46.3 ± 7.2 s).

After Sessions 1–3 (Day 30), SIB decreased to three to five episodes daily, aggression decreased to three to four episodes weekly, and restraint requirements decreased to one to two episodes weekly. Sleep deteriorated to 3–4 h each day (**Table 1**).

After Session 6 (Day 37), transient exacerbation was observed: the VRAS score increased to 22, SIB escalated to six to eight episodes daily, and aggression occurred four to five times a week (**Table 1**). A multidisciplinary review revealed no new deficits or complications. Treatment was continued based on (1) 11 years of pharmacotherapy refractoriness (2); the absence of complications (3); adequate seizure parameters (4); literature supporting continuation during mid-treatment fluctuations; and (5) baseline SIB severity. Enhanced monitoring was implemented with the guardian’s reaffirmed informed consent. As the patient remained unable to provide assent owing to his severe intellectual disability, the guardian continued to act as the sole decision-maker in accordance with the least-restrictive, best-interest principle.

By Session 12 (Day 76), a marked improvement was achieved (**Table 1**, **Figure 2**). The VRAS score decreased from 18 to 7 (61.1% reduction). SIB resolved completely, with complete tissue healing (**Figure 1**). The BPRS score decreased to 47 (36.5% reduction), and the ADL score decreased to 34 (26.1% improvement in functional independence). All maladaptive behaviors were eliminated; aggression and the need for physical restraints were reduced to zero.

The patient remained hospitalized throughout (Days 1–106). During the post-MECT maintenance phase, the patient received risperidone (2 mg/day) and valproic acid (1,000 mg/day). At the 30-day follow-up (Day 106), treatment gains were sustained and further enhanced. The VRAS score persisted at 7, and the BPRS score declined to 35 (52.7% reduction from baseline). The ADL score improved to 30 (34.8% improvement from baseline). All maladaptive behaviors remained in complete remission. Despite sustained behavioral improvements, sleep returned to baseline levels (4–5 h/night), suggesting a potentially distinct sleep pathophysiology (**Table 1**, **Figure 2**). No serious adverse events were observed. **Table 2** provides a comprehensive treatment and outcome timeline, including MECT session parameters, adverse events, behavioral outcomes, and scale scores at each assessment time point. **Table 3** provides complete information on all medication changes throughout hospitalization, including the pre-MECT pharmacotherapy trial and MECT-period adjustments.

**Table 2:** Comprehensive treatment and outcome timeline.

Hospitalization day	Phase	Adverse events	SIB (times/day)	Aggression (times/week)	Restraints (times/week)	Sleep (h/night)	VRAS	BPRS	ADL
Days 1–26	Pre-MECT pharmacotherapy	None	5–6	5–7	3–4	4–5	–	–	–
Day 27 (Baseline)	MECT initiation (Session 1)	None	5–6	5–7	3–4	4–5	18	74	46
Day 30	Active MECT (Session 3)	None	3–5	3–4	1–2	3–4	–	–	–
Day 37	Active MECT (Session 6)	Transient exacerbation	6–8	4–5	3–4	3–4	22	–	–
Day 55	Active MECT (Session 9)	None	0	1–2	0	4–5	–	–	–
Day 76	Session 12 (Post-treatment)	None	0	0	0	5–6	7	47	34
Day 106	30 Days Post-MECT	None	0	0	0	4–5	7	35	30

Behavioral frequencies were documented by nursing staff via standardized 24-hour observation; recording methodology is detailed in the Diagnostic Investigations section. BPRS and ADL were assessed at pre-specified timepoints only (baseline, post-treatment, and follow-up); VRAS was additionally assessed at Session 6 due to observed transient behavioral exacerbation. Medication details and MECT technical parameters are described in the Treatment Protocol section. –, not assessed at that timepoint. MECT, modified electroconvulsive therapy; SIB, self-injurious behavior; VRAS, Violence Risk Assessment Scale; BPRS, Brief Psychiatric Rating Scale; ADL, Activities of Daily Living Scale.

**Table 3:** Medication timeline in relation to MECT sessions and clinical assessments.

Hospitalization day	Phase	Valproic acid (mg/day)	Risperidone (mg/day)	Olanzapine (mg/day)
Days 1–10	Pre-MECT (initial trial)	1000	–	20
Days 11–26	Pre-MECT (regimen change)	1000	3	–
Day 27	MECT Initiation (Session 1)	0 ↓	2 ↓	–
Days 28–76	Active MECT (Sessions 2–12)	0	2	–
Day 76	Post-MECT (Session 12 completed)	1000 ↑	2	–
Days 76–106	Post-MECT Maintenance	1000	2	–

Days 1–10 represent the initial pharmacotherapy trial (olanzapine 20 mg/day plus valproic acid 1000 mg/day); inadequate clinical response prompted a regimen change to risperidone plus valproic acid from Day 11. On Day 27, risperidone was reduced to 2 mg/day, and valproic acid was discontinued for the duration of MECT to minimize seizure threshold interference. Valproic acid was reintroduced at 1000 mg/day upon completion of MECT on Day 76. ↓ = dose reduction or discontinuation; ↑ = dose reintroduction; – = not administered. MECT, modified electroconvulsive therapy.

## Discussion

This case suggests that MECT may contribute to the reduction of treatment-refractory SIB in individuals with ASD and comorbid severe ID, while appearing well tolerated in this context. After 11 years of pharmacotherapy failure, stepwise MECT achieved complete SIB resolution (from 5–6 episodes daily to zero), with substantial reductions in violence risk (VRAS score: 61.1%) and psychiatric symptoms (BPRS score: 52.7% at follow-up), in addition to improved daily functioning and independence (ADL score: 34.8% improvement at follow-up). These benefits persisted and further improved at the 30-day post-MECT evaluation while the patient remained hospitalized.

The pathophysiology of SIB in ASD-ID populations involves dysregulation of endogenous opioid systems (14), multi-neurotransmitter disturbances (15), anomalies in sensory processing (17), and alterations in the somatosensory cortex and thalamocortical pathways (18). Single-target medications are often insufficient because of these multilayered pathological substrates. Multi-neurotransmitter modulation (19), brain-derived neurotrophic factor (BDNF)-mediated neuroplasticity (20), and optimization of prefrontal-limbic connectivity (21, 22) are well-established mechanisms of MECT in mood disorders and catatonia. However, whether these mechanisms directly mediate SIB reduction in ASD-ID populations remains unclear and requires empirical investigation. Future neurobiological studies using functional neuroimaging, electrophysiological biomarkers, and neurochemical assays are warranted to elucidate the specific therapeutic targets of MECT in this population.

These findings are broadly consistent with existing MECT literature in demonstrating meaningful symptomatic improvement in patients with ASD and severe, treatment-refractory SIB. Nonetheless, important distinctions merit discussion. Prior reports predominantly involved catatonia as the primary indication for ECT. Wachtel et al. (23) described 22 patients with ASD and SIB interpreted as agitated catatonia, and Haq et al. (24) reported two adolescents with autism and catatonia. In contrast, the present case involved an adult patient with severe ID and 11-year pharmacotherapy-refractory SIB without documented catatonia, representing a clinically distinct subgroup. Regarding the measurement approach, prior studies relied predominantly on the Clinical Global Impression (CGI) scale or its Improvement subscale (CGI-I), whereas a recent meta-analytic review by Herrera-Pino et al. (25) highlighted the Overt Aggression Scale (OAS) and Modified Overt Aggression Scale (MOAS) as the most empirically supported instruments for this population. The present case used a multidimensional framework consisting of behavioral frequency counts, VRAS scores, BPRS scores, ADL scores, and photographic documentation, providing enhanced granularity of the outcomes. Most importantly, the patient achieved complete resolution of SIB without clinically apparent catatonic features noted on routine clinical observation, although formal catatonia screening was not performed. This observation raises the possibility that the therapeutic mechanisms of MECT may extend beyond catatonia resolution. This finding is contrary to the prior literature. Furthermore, SIB and aggression were systematically tracked using quantified frequency data and multi-scale psychiatric assessments, which offered a more reproducible outcome framework than previous CGI-based global ratings. Collectively, this case expands the body of data by including a non-verbal adult with severe ID and catatonia-independent, pharmacotherapy-refractory SIB, a profile that is underrepresented in the literature. Additionally, it proposes a methodological template for more rigorous outcome documentation in future studies.

Despite these limitations, the existing literature on maintenance strategies offers clinically useful guidance. Haq and Ghaziuddin (24) demonstrated sustained SIB control over approximately 12 months using maintenance ECT at progressively extended and ultimately monthly intervals. In contrast, Wachtel et al. (23) reported long-term maintenance ECT in pharmacotherapy-refractory cases, with follow-up extending up to 12 years in selected patients, although generalizability was limited by variable protocols. A structured cross-study comparison (**Table 4**) further showed substantial heterogeneity in study design, follow-up duration, and outcome sustainability. Wachtel et al. (26) and Smith et al. (16) provided no post-discharge follow-up, leaving long-term durability uncharacterized. However, Haq and Ghaziuddin (24) provided the most robust evidence regarding durability. Collectively, long-term relapse patterns, time-to-recurrence, and the comparative durability of pharmacological and ECT-based maintenance strategies remain poorly defined, and no prospective study has systematically addressed these questions in ASD-ID populations. In the present case, post-MECT pharmacological maintenance with risperidone (2 mg/day) combined with valproic acid (1,000 mg/day) was associated with sustained and further enhanced behavioral remission across all outcome domains by Day 106 following 12 acute sessions over 7 weeks. However, community-based durability has not been evaluated, and the contribution of a structured inpatient environment cannot be excluded. Considering the patient’s 11-year history of pharmacotherapy-refractoriness, symptom recurrence after discharge remains possible. Therefore, maintenance ECT may be considered as a contingency strategy should pharmacological control prove insufficient. Future studies should systematically evaluate indications, optimal interval schedules, and discontinuation criteria for ECT maintenance, incorporating extended follow-up (≥ 6 months) and community-based assessments to better characterize long-term treatment durability in this population.

**Table 4:** Structured comparison of key studies on ECT for self-injurious behavior in ASD: study design, follow-up duration, and outcome sustainability.

Study	Design	N	Population	Primary indication	ECT sessions (Acute)	Follow-up duration	Outcome sustainability
Wachtel et al., 2010 (26)	Case report	1	Adult male with ASD, catatonia, SIB	Catatonia/SIB	Not specified	Not reported	Improvement during hospitalization; long-term durability not reported
Haq & Ghaziuddin, 2014 (24)	Case series	2	Adolescents with ASD and catatonia	Catatonia/SIB/aggression	10–12 sessions	Up to 12 months	Sustained control with maintenance ECT; good tolerability
Wachtel, 2019 (23)	Retrospective case series	22	ASD patients, catatonia with SIB	Catatonia/SIB	Variable	Up to 12 years (in selected patients, variable protocols)	Long-term control achieved in subset via maintenance ECT; unstandardized protocols limit generalizability
Smith et al., 2024 (16)	Single-site retrospective analysis	32	ASD and/or ID patients	SIB and behavioral dysregulation	Variable	Not reported	Significant acute response; no post-discharge follow-up data reported
Present case	Case report	1	Adult male with ASD and severe ID, with 11-year pharmacotherapy-refractory SIB	SIB (non-catatonic)	12 sessions over 7 weeks	30 days post-MECT (Day 106)	Gains sustained and further enhanced at Day 106; community durability unknown

ECT, electroconvulsive therapy; MECT, modified electroconvulsive therapy; ASD, autism spectrum disorder; ID, intellectual disability; SIB, self-injurious behavior; N, number of cases. “Not reported” indicates no post-discharge follow-up data were available in the original publication. All session numbers refer to the acute treatment course only; maintenance ECT protocols are described narratively in the Outcome Sustainability column.

The pragmatic stepwise protocol (3→2→1 sessions weekly) balanced acute control and feasibility. Notably, transient exacerbation occurred after Session 6 (VRAS score: 18→22; SIB frequency: 5–6→6–8 episodes daily), but continuation of treatment resulted in complete resolution by Session 12. This mid-treatment worsening may reflect neuroadaptation observed during MECT for depression, cumulative propofol effects, elevated seizure thresholds, or psychosocial stressors. Adequate seizure parameters, the absence of medical problems, baseline refractoriness, and research supporting therapy through mid-course variations served as guidelines for continuation.

Unlike prior reports based on single-scale assessment, the present case used a multidimensional outcome framework. Primary behavioral outcomes comprised direct frequency counts of SIB (episodes/day), aggression (episodes/week), and physical restraint episodes (episodes/week), systematically documented by nursing staff under continuous around-the-clock monitoring. VRAS, BPRS, and ADL scores served as validated secondary instruments reflecting violence risk, overall psychiatric symptom burden, and functional independence, respectively. The ADL assessment employed in this study was the 14-item Chinese Activities of Daily Living Scale (C-ADL; range 14–56), a validated observer-rated instrument widely used in Chinese institutionalized populations with significant functional impairment. Unlike internationally standardized instruments such as the Barthel Index, Katz Index, and Lawton Instrumental ADL scale, the Chinese ADL Scale employs an inverse scoring system in which lower scores indicate greater functional independence (27). A total score below 16 is considered functionally normal, whereas scores above 16 indicate varying degrees of functional decline, with higher scores reflecting greater dependence. The VRAS was specifically selected for its aggression-related items, enabling standardized tracking of both self-directed and other-directed harmful behaviors. The Chinese version of the VRAS (VRAS-C) has been validated in Chinese psychiatric inpatients, demonstrating good scorer reliability (intraclass correlation coefficient [ICC] = 0.80), high internal consistency (Cronbach’s α = 0.921), satisfactory split-half reliability (0.906), and adequate item-total correlations (r = 0.246–0.849) (28). Photographic recording provided additional objective evidence of wound healing throughout the treatment course. Collectively, this provided a realistic approach to outcome monitoring by capturing treatment dynamics, including mid-treatment fluctuations. Pre-MECT treatment with risperidone (3 mg/day) and valproic acid (1,000 mg/day) for 16 days was ineffective, whereas post-MECT maintenance with risperidone (2 mg/day) and valproic acid produced sustained control. This is an interesting pharmacological observation that warrants cautious interpretation. This finding may reflect MECT-induced neuroplastic changes that reduced pharmacological thresholds, the resolution of MECT-responsive symptom components, or synergistic effects between valproic acid and post-MECT neurobiological changes. Concurrent valproic acid treatment represents a significant confounding factor. Although pre-MECT treatment demonstrated insufficient efficacy, its contribution to post-MECT stabilization cannot be ruled out. The difficulty in distinguishing between distinct treatment effects underscores the limitations of this naturalistic design. It is crucial to conduct controlled studies with systematic medication withdrawal and rechallenge strategies.

Despite severe ID, resolved tuberculosis, and general anesthesia, this case provides safety data on medical complexity: 12 MECT sessions completed without serious adverse events. There was only a brief decrease in sleep duration, which improved to 5–6 h per night by treatment completion and returned to baseline levels at follow-up. However, severe ID precluded rigorous cognitive assessment, making it challenging to systematically evaluate MECT-related cognitive effects. Although no clinically apparent deterioration occurred, this represents a methodological limitation and highlights the need for ID-adapted cognitive tools.

Several limitations warrant acknowledgment beyond the constraints inherent to a single-case design. First, because of the patient’s severe ID, lack of verbal communication, and acute behavioral dysregulation, SIB- and aggression-specific instruments, such as the Behavior Problems Inventory (BPI-01) and OAS, were not formally administered. Notably, the absence of the metacognitive ability necessary for first-person functional attribution also prohibited the use of self-report-dependent measures, such as the Inventory of Statements About Self-Injury (ISAS). In addition to general psychiatric scales, future studies should prioritize informant-based or observational SIB instruments (e.g., BPI-01) that have been validated for non-verbal individuals with severe ID. Second, concurrent pharmacotherapy precludes the definitive attribution of the improvement solely to MECT, with observed benefits likely reflecting combined treatment effects. Third, formal catatonia screening using a validated instrument, such as the Bush-Francis Catatonia Rating Scale (BFCRS), was not conducted at any point during the clinical course. Consequently, the presence or absence of subclinical or overt catatonic features cannot be definitively established. Future case reports and studies should incorporate standardized catatonia assessments to better delineate the relationship among catatonia, SIB, and MECT response in this population. Fourth, it is not possible to evaluate treatment durability or community relapse risk using the 30-day post-MECT follow-up conducted within a structured inpatient setting. This limitation reflects a broader gap in the ASD-ECT literature. Smith et al. (16) did not report longitudinal follow-up data. Wachtel et al. (23) described multi-year maintenance ECT in selected patients; nonetheless, the protocols were non-standardized. Future studies should incorporate extended follow-up (≥ 6 months) with community-based assessment phases and prospectively evaluate the indications and optimal times of maintenance ECT in this population. Furthermore, several contextual factors limit causal attribution. The highly structured 24-hour inpatient environment, with continuous nursing supervision and behavioral containment, may independently reduce SIB. The patient’s reunification with his family after 11.5 years of separation—a major psychosocial transition occurring concurrent with MECT—represents an additional uncontrolled variable that may have independently influenced behavioral outcomes. In addition, given the exceptional severity of the pre-treatment baseline, regression to the mean cannot be excluded as a partial contributor to the observed improvement. Fifth, mechanistic insights are limited by the absence of neurobiological investigations. Finally, generalizability to larger ASD-ID populations may be limited by the patient’s unique psychosocial history (11.5 years of family separation), medical complexity, and specific behavioral phenotype.

In conclusion, this case suggests that MECT may facilitate short-term control of treatment-refractory SIB in patients with ASD and severe ID within a highly structured inpatient setting. Long-term follow-up is needed to determine whether these improvements persist after the transition to community settings. However, single-case evidence is inherently insufficient to inform standard clinical practice. Multicenter randomized controlled trials, extended follow-up (≥ 6 months), neuroimaging and biomarker studies, and standardized implementation protocols are important next steps for future research.

## Data Availability

The original contributions presented in the study are included in the article/**Supplementary Material**. Further inquiries can be directed to the corresponding authors.
