# Supplementary material for: Modified electroconvulsive therapy for treatment-resistant self-injurious behavior in autism spectrum disorder with severe intellectual disability: a case report
Source: Front Psychiatry. 2026 Jun 17;17:1800695. doi: 10.3389/fpsyt.2026.1800695 (PMC13321460; doi:10.3389/fpsyt.2026.1800695)
Supplement: Supplementary file 1 [file Supplementaryfile1.docx]

Supplementary Table 1: Baseline Laboratory and Diagnostic Findings

| Category | Test | Result | Reference Range |
| --- | --- | --- | --- |
| Hematology | Complete blood count | WBC: 5.89×10⁹/L; RBC: 4.78×10¹²/L; Hb: 134 g/L; PLT: 285×10⁹/L | WBC: 4.0–10.0×10⁹/L; RBC: male, 4.0–5.5×10¹²/L, female, 3.5–5.0×10¹²/L; Hb: male, 120–160 g/L, female, 110–150 g/L; PLT: 100–300×10⁹/L |
| Hepatic function | Hepatic function panel | ALT: 25 U/L; AST: 27 U/L; TB: 8.1 μmol/L; ALB: 49.2 g/L | ALT: 0–40 U/L; AST: 0–37 U/L; TB: 1.7–21.0 μmol/L; ALB: 34.0–48.0 g/L |
| Renal function | Renal function panel | BUN: 4.0 mmol/L; Cr: 66 μmol/L; UA: 414.0 μmol/L | BUN: 1.7–8.3 mmol/L; Cr: male, 59–104 μmol/L, female, 45–84 μmol/L; UA: male, 208–428 μmol/L, female, 155–357 μmol/L |
| Serology | Infectious disease screening | Negative | HIV Ab, HBsAg, HCV Ab, and syphilis Ab: all negative |
| Microbiology | Sputum AFB smear | Negative | Negative |
|  | Sputum culture | Negative | Negative |
| Electrocardiography | 12-lead ECG | Sinus rhythm: 75 bpm; PR interval: 0.13 s; QRS duration: <0.09 s | Sinus rhythm: 60–100 bpm; PR interval: 0.12–0.20 s; QRS duration: <0.12 s |
| Imaging | Chest CT (August 6, 2025) | Left upper lobe apicoposterior segment micronodule (approximately 3 mm in diameter); minimal fibrotic strands in right middle lobe consistent with old tuberculosis | No nodules, masses, or infiltrates; clear lung parenchyma |
| Neurophysiology | Electroencephalography | No epileptiform discharges | Normal background activity; no epileptiform discharges |

Abbreviations: WBC, white blood cell count; RBC, red blood cell count; Hb, hemoglobin; PLT, platelet count; ALT, alanine aminotransferase; AST, aspartate aminotransferase; TB, total bilirubin; ALB, albumin; BUN, blood urea nitrogen; Cr, creatinine; UA, uric acid; AFB, acid-fast bacilli; Ab, antibody; HIV, human immunodeficiency virus; HBsAg, hepatitis B surface antigen; HCV, hepatitis C virus; ECG, electrocardiogram; CT, computed tomography.
